# Coincident Correlation between Vibrational Dynamics and Primary Relaxation of Polymers with Strong or Weak Johari-Goldstein Relaxation

**DOI:** 10.3390/polym12040761

**Published:** 2020-03-31

**Authors:** Antonio Tripodo, Francesco Puosi, Marco Malvaldi, Simone Capaccioli, Dino Leporini

**Affiliations:** 1 Dipartimento di Fisica “Enrico Fermi”, Università di Pisa, Largo B.Pontecorvo 3, I-56127 Pisa, Italy; antonio.tripodo@df.unipi.it (A.T.); francesco.puosi@df.unipi.it (F.P.); marcoampelio@hotmail.com (M.M.); simone.capaccioli@unipi.it (S.C.); 2 Istituto per i Processi Chimico-Fisici-Consiglio Nazionale delle Ricerche (IPCF-CNR), via G. Moruzzi 1, I-56124 Pisa, Italy

**Keywords:** polymer melt, primary and secondary relaxations, Johari-Goldstein relaxation, bond reorientation, vibrational dynamics, molecular-dynamics simulations

## Abstract

The correlation between the vibrational dynamics, as sensed by the Debye-Waller factor, and the primary relaxation in the presence of secondary Johari-Goldstein (JG) relaxation, has been investigated through molecular dynamics simulations. Two melts of polymer chains with different bond length, resulting in rather different strength of the JG relaxation are studied. We focus on the bond-orientation correlation function, exhibiting higher JG sensitivity with respect to alternatives provided by torsional autocorrelation function and intermediate scattering function. We find that, even if changing the bond length alters both the strength and the relaxation time of the JG relaxation, it leaves unaffected the correlation between the vibrational dynamics and the primary relaxation. The finding is in harmony with previous studies reporting that numerical models *not showing secondary relaxations* exhibit striking agreement with experimental data of polymers also where the presence of JG relaxation is known.

## 1. Introduction

If polymers and liquids avoid crystallization during cooling or compression, they freeze into a microscopically disordered solid-like state, a glass [1]. On approaching the glass transition, molecular rearrangements occur via both the primary mode, referred to as structural or *α* relaxation, and the faster secondary (*β*) processes as evidenced by mechanical, electrical, and thermal properties of materials [2,3,4]. Although it has been at the focus of a large number of phenomenological and theoretical studies, as well as of experiments and simulations [4,5,6,7,8,9,10], there is still no definitive microscopic description available for the *β* relaxation. In particular, there is a special class of secondary relaxations involving translation or reorientation of the molecular unit as a whole, well different from the trivial ones, related to intramolecular degrees of freedom, such as the motion of pendant groups in polymers. This special class of *β* processes, called Johari Goldstein (JG) to honor the researchers that first noticed it [5], is universal in glass formers [4,6,7] and has been shown [6] to have strong relation to structural *α*-relaxation, being both governed by intermolecular interactions. A number of experimental results indicate that this *β* JG relaxation is sensitive to the thermodynamic variables underlying the glass transition [4,7], mimicking the *α* relaxation, being strongly pressure dependent and showing the invariance of the ratio *τ_α_*/*τ_β_* to variations of pressure and temperature, keeping *τ_α_* constant. *β* JG, for these reasons, can be considered as the precursor to structural relaxation, having a slower dynamics due to cooperativity involving many body dynamics [6,8].

The purpose of the present paper is to investigate if the presence of well resolved secondary relaxation could disprove the universal correlation between the vibrational dynamics and the primary relaxation observed by experiments and numerical simulations in highly viscous liquids [11], for a recent mini review see Reference [12]. We are motivated by the fact that the timescale of the secondary relaxation (${\bar{\tau }}_{s}$) is *intermediate* between the vibrational timescale (t*), a few picoseconds, and the structural relaxation time of the primary relaxation (${\bar{\tau }}_{p}$) which reaches about hundreds of seconds close to the glass transition:

(1) $$t^{*}\ll {\bar{\tau }}_{s}<{\bar{\tau }}_{p}$$

The exact definitions of the symbols in Equation (1) will be given in Section 3. Here, we are interested in the secondary relaxation observed in the *main chain* of *linear* polymers without side groups. This class fits the definition of Johari-Goldstein (JG) relaxation [7]. There is consensus that in linear chains the JG process involves local motion of the polymer backbone. 

Recently, the investigation of JG relaxation by using MD simulations has been facilitated by the development of coarse-grained models of linear polymers having fixed bond length and bond angles constrained to 120° [13,14,15]. We also mention studies of the JG relaxation in asymmetric diatomic molecules [15,16].

## 2. Model and Numerical Methods

**Figure 1:**
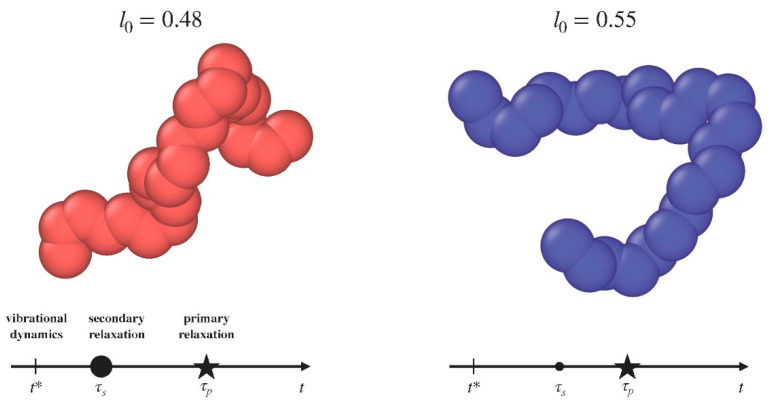
Pictorial view of the two kind of polymeric chains under consideration. *L*_0_ denotes the bond length. The time axis signal, in a qualitative way, the location of the dynamical processes of interest. The size of the dot of the secondary relaxation is proportional to the strength of the relaxation. The exact definition of the symbols is given in Section 3.

We investigate two coarse-grained models of linear polymer chains. The melts are constituted by *N_c_* = 512 linear chains made of *M* = 25 monomers each, resulting in a total number of monomers *N* = 12800. Non-bonded monomers at a distance *r* interact via a Lennard-Jones potential:

(2) $$U^{LJ}\left(r\right)=\varepsilon \left[{\left(\frac{\sigma^{*}}{r}\right)}^{12}-2{\left(\frac{\sigma^{*}}{r}\right)}^{6}\right]$$

where σ* = 2^1/6^*σ* is the minimum of the potential, $U^{LJ}\left(r=\sigma \right)=-\epsilon$. The potential is truncated at *r* = *r_c_* = 2.5*σ* for computational convenience. Adjacent bonded monomers interact with each other via the harmonic potential *U^bond^*(*r*) = *k_bond_*(*r* − *l*_0_)^2^, where the constant *k_bond_* is set to $2000\epsilon /\sigma^{2}$ to ensure high stiffness. We consider two distinct cases, corresponding to rest bond length *l*_0_ set to either *l*_0_ = 0.48*σ* or *l*_0_ = 0.55*σ*. The rationale behind our choice of the two bond lengths relies on the finding that previous Molecular-Dynamics (MD) simulations of the present model [14], investigating the rotational dynamics, revealed the steep *increase* of the separation between the primary and the JG relaxations by *decreasing* the bond length below $l_{0}^{*}=0.5\sigma$ (corresponding to 2Å in Figure 3 of Reference [14]). Therefore, one anticipates that the JG relaxation is much more apparent if *l*_0_ = 0.48*σ* with respect to *l*_0_ = 0.55*σ*. A bending potential *U^bend^*(*α*) = *k_bend_* (cos *α* − cos *α*_0_)^2^, with *kbend* = 2000ϵ/*σ*^2^ and *α*_0_ = 120°, is introduced to maintain the angle *α* formed by two consecutive bonds nearly constant (see Figure 1 for typical chain conformations) [17].

All the data presented in this work are expressed in reduced MD units: length in units of *σ*, temperature in units of *ϵ*/*k_B_*, where *k_B_* is the Boltzmann constant, and time in units of *τ_MD_* = (*mσ*^2^/*ϵ*)^1/^^2^. We set *σ* = 1, *ϵ* = 1, *m* = 1 and *k_B_* = 1.

Simulations were carried out with the open-source software LAMMPS [18,19]. Equilibration runs were performed at constant number of monomers *N*, constant vanishing pressure *P* = 0 and constant temperature *T* (*NPT* ensemble) [20]. For each state the equilibration lasted at least for 3*τ_ee_*, being *τ_ee_* the relaxation time of the end-to-end vector autocorrelation function [21,22,23,24,25,26,27,28]. Production runs have been performed within the *NVT* ensemble (constant number of monomers *N*, constant volume *V* and constant temperature *T*). Additional short equilibration runs were performed when switching from *NPT* to *NVT* ensemble. No signatures of crystallization were observed in all the investigated states.

As a final remark, we point out that our adoption of a monodisperse polymer model where all the chains have equal length is not expected to limit our conclusions. In fact, the vibrational scaling collapses in a single master curve both the MD results of monodisperse polymer models and the experimental data on polymers which, expectedly, refer to polydisperse systems [11].

## 3. Results and Discussion

### 3.1. Bond Reorientation

Since in linear chains the JG process involves local motion of the polymer chain backbone [4,7], we focus on the most elementary relaxation process, that is, the reorientation of the bond linking two adjacent monomers of the same chain. Let us define a suitable average bond correlation function *C*(*t*) [29]. To this aim, we consider the unit vector along the *m*-th bond of the *n*-th chain at time *t*:

(3) $$b_{m,n}\left(t\right)=\frac{1}{l_{0}}\left(r_{m,n}\left(t\right)-r_{m+1,n}\left(t\right)\right)$$

where *r*_*m*,*n*_ (*t*) denotes the position of the *m*-th monomer in the *n*-th chain at time *t*. We define the correlation function *C*(*t*):

(4) $$C\left(t\right)=\frac{1}{N_{c}}\frac{1}{M-1}\overset{N_{c}}{\underset{n=1}{\sum }}\overset{M-1}{\underset{m=1}{\sum }}\langle b_{m,n}\left(t\right)\cdot b_{m,n}\left(0\right)\rangle$$

The brackets 〈⋯〉 denote a suitable ensemble average. *C*(*t*) decreases in time (by definition *C*(0) = 1), finally vanishing at long times when the bond orientation has explored all the unit sphere.

**Figure 2:**
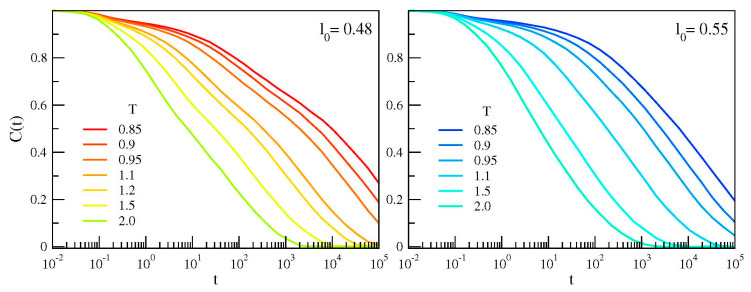
Temperature dependence of the bond correlation function of the chains with different bond length. If *l*_0_ = 0.48, a two-step decay—evidencing two distinct relaxations—is observed.

Figure 2 plots the correlation function *C*(*t*) for the two polymer melts with chains having different bond length. In agreement with previous studies [13,14], it is seen that the chains having shorter bond length exhibits a characteristic two-step decay, signaling the presence of two distinct relaxation processes.

To gain more insight, the correlation function *C*(*t*) was analyzed under the assumption that it is represented by the weighted sum of two components, that is, a fast secondary process and a slower primary one:

(5) $$C\left(t\right)=A_{p}f_{p}\left(t\right)+A_{s}f_{s}\left(t\right)$$

where *f_p_*(*t*) and *f_s_*(*t*) are two decaying functions with amplitudes *A_p_* and *A_s_*, respectively. The explicit form of *f_i_*(*t*) is taken as a stretched exponential:

(6) $$f_{i}\left(t\right)=\exp\left[-{\left(\frac{t}{\tau_{i}}\right)}^{\beta_{i}}\right],\;i=p,s$$

where *τ_i_* denotes the relaxation time and *β_i_* the stretching exponent (as normally *β_i_* ≤ 1). We fit the MD data concerning the correlation function *C*(*t*) with Equation (5) excluding the time window *t* ≤ $\hat{t}$ where the decay of the function is controlled by the ballistic motion of monomers (0.6 ≤ $\hat{t}$ ≤ 1 with $\hat{t}$ decreasing slightly by increasing *T*).

The analysis in terms of a sum of two stretched exponential decays, Equation (5) and Equation (6), conforms with most studies of primary and secondary relaxations in MD simulation [13,14,15,30,31] and in some experiments done in time domain [32,33]. Then, it facilitates the comparison between our results to those present in literature.

We have considered also the use of alternative functions to fit the secondary relaxation. For instance, we tested the Mittag Leffer function (with 2 fitting parameters like the stretched exponential), corresponding to the time-domain counterpart of the Cole-Cole function and usually used in studies of dielectric spectroscopy to describe the secondary process [34,35]. Yet, no significant differences have been found in terms of relaxation times and strengths with respect to the analysis with the sum of stretched exponentials.

**Figure 3:**
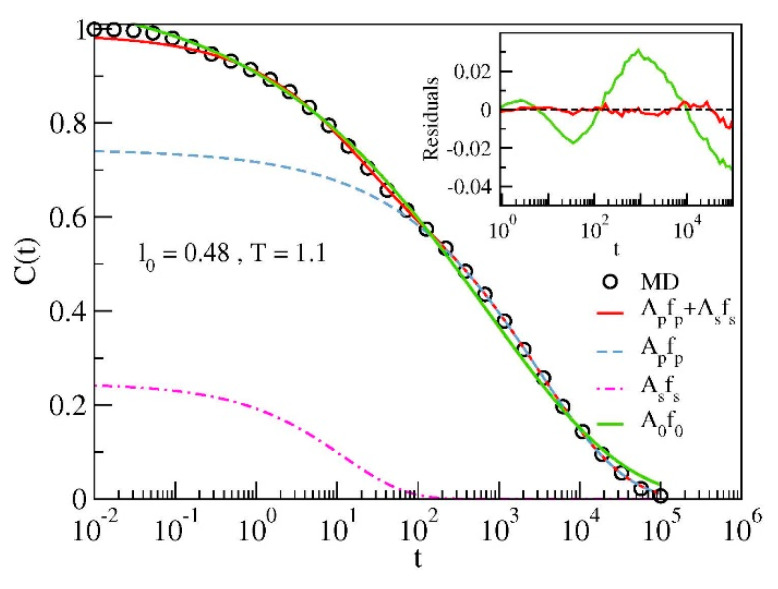
Illustrative example of the best-fit results via the double-relaxation function Equation (5). The best-fit with the single-relaxation function *A*_0_ *f*_0_ with *f*_0_ in the form of Equation (6) is also shown. Inset: residuals of the best-fit with the double- and single- relaxation functions.

Figure 3 shows the result of the best-fit of *C*(*t*) with Equation (5) and Equation (6) for a representative state of the model. For comparison, the best-fit with a single stretched exponential decay is also shown. The residuals *C^MD^*(*t*) − *C*^fit^(*t*) plotted in the inset of Figure 3 prove the poor performance of the single-relaxation curve to fit the MD data.

**Figure 4:**
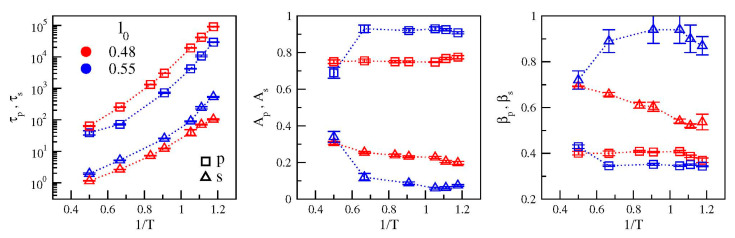
Plots of the temperature dependence of the best-fit parameters by using Equation (5) and Equation (6) (color codes as in Figure 1). From left to right: apparent relaxation times, relaxation strengths and stretching exponents.

Figure 4 presents the temperature dependence of the parameters extracted from the best-fit of data with Equation (5) and Equation (6). It is seen that reducing the bond length has two major effects: (i) *increased* separation of the relaxation times *τ_s_* and *τ_p_* (ii) *enhanced* amplitude of the secondary relaxation with respect to the primary one. It is also worth noting that the primary relaxation is less stretched in the model where the secondary relaxation is more apparent (*l*_0_ = 0.48) with respect to the case in which it is weak (*l*_0_ = 0.55). The differences are small but significant, that is, larger than the errors on the stretching exponents. Stretched relaxation is usually associated with the presence of dynamical heterogeneities, namely the spatial distribution of mobilities, which may differ of orders of magnitude in regions only a few nanometers away. In this framework, our results suggest that the presence of a not negligible secondary relaxation process slightly decreases the degree of dynamical heterogeneity of the system. We plan to address this aspect in future works.

To get rid of the temperature dependence of the stretching exponent β, we define the average relaxation time for the primary and the secondary relaxations, ${\bar{\tau }}_{p}$ and ${\bar{\tau }}_{s}$ respectively, as the time-integral of the corresponding relaxation function (*f_p_* and *f_s_*) leading to

(7) $${\bar{\tau }}_{i}=\frac{\tau_{i}}{\beta_{i}}\Gamma \left(\frac{1}{\beta_{i}}\right),\;i=p,s$$

**Figure 5:**
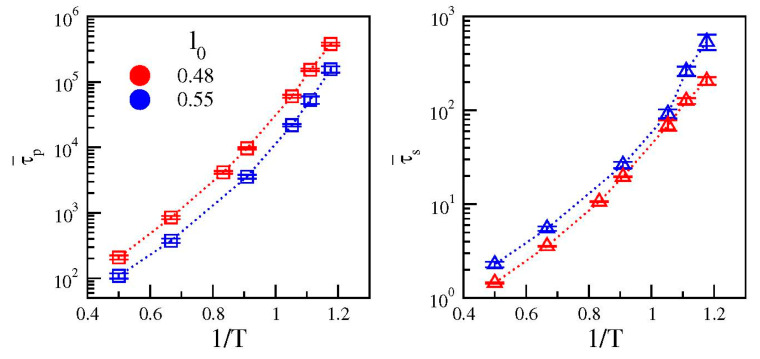
Arrhenius plots of the average relaxation times of the primary (${\bar{\tau }}_{p}$, left) and secondary (${\bar{\tau }}_{s}$, right) relaxations. Color code as in Figure 1.

The temperature dependence of the average relaxation times of the two polymer models are shown in Figure 5. For both systems the primary relaxation time (left panel in Figure 5) grows more than three orders of magnitude in the range of temperature from *T* = 2.0 to *T* = 0.85, exhibiting a non-Arrhenius upward concavity which is typical of fragile glassformers [36]. Conversely, the growth of the secondary relaxation time (right panel in Figure 5) is less pronounced (approximately two and a half decades in the same temperature range) and the deviations from an Arrhenius behavior are weaker, in particular for the system with stronger secondary relaxation (*l*_0_ = 0.48, red symbols).

### 3.2. Monomer Mobility

**Figure 6:**
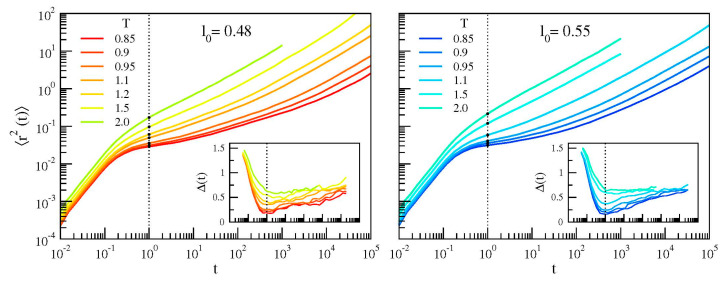
Monomer mean square displacement (MSD) of the two polymer melts with chains having bond lengths *l*_0_ = 0.48 (left) and *l*_0_ = 0.55 (right). Inset: corresponding MSD slope ∆(*t*), Equation (9). The vertical dashed lines mark the time t^*^ ≈ 1 where ∆(*t*) reaches the minimum, locating the time where caging is more effective. t^*^ is found to independent of both the system and its physical state. The black circles indicate the values of $\langle u^{2}\rangle$, Equation (10).

With the aim of further characterizing the dynamics of the two polymer melts with chains having distinct bond lengths, we now consider the monomer mobility, as quantified by the mean square displacement (MSD)

(8) $$\langle r^{2}\left(t\right)\rangle =\frac{1}{N}\overset{N}{\underset{i=1}{\sum }}\langle {\|x}_{t}\left(t\right)-x_{i}{\left(0\right)\|}^{2}\rangle$$

where *x_i_* (*t*) is the position of the *i*-th monomer at time *t*. Figure 6 shows MSD curves for the two systems at all the investigated temperatures. At very short times (ballistic regime) MSD increases according to $\langle r^{2}\left(t\right)\rangle ≅\left(3k_{B}T/m\right)t^{2}$. At later times a quasi-plateau region becomes apparent when the temperature is lowered. This signals the increased trapping of a particle in the cage of its neighbors. Once escaped from the cage, due to the presence of the chain connectivity, the monomers undergo a sub diffusive motion $\langle r^{2}\left(t\right)\rangle \propto t^{\delta }$ with *δ <* 1 (Rouse regime) [21]. At very long times, monomers displace in a diffusive way (*δ* = 1). Diffusion is hardly seen in our simulations since, due to the length of the chains, it occurs at the limit of the accessible timescales.

### 3.3. Cage Dynamics and Correlation with Primary and Secondary Relaxations

To identify a characteristic time-scale of the cage dynamics we consider the slope of MSD in the log-log representation

(9) $$∆\left(t\right)\equiv \frac{\partial \log\langle r^{2}\left(t\right)\rangle }{\partial \log t}$$

The slope ∆(*t*) is plotted in the insets of Figure 6. ∆(*t*) tends to 2 at very short times, due to the ballistic motion. Then, it drops to a minimum value signaling effective trapping in the cage. Later, ∆(*t*) increases—staying less than 1 due to the Rouse sub-diffusion—with a progressive approach to the unit value characterizing the diffusive regime. The minimum occurs at $t^{*}\approx 1$ independently of both the system and its physical state. It offers a convenient definition of the monomer Debye-Waller (DW) factor $\langle u^{2}\rangle$ as the MSD at $t^{*}$ [11,12]:

(10) $$\langle u^{2}\rangle \equiv \langle r^{2}\left(t=t^{*}\right)\rangle$$

**Figure 7:**
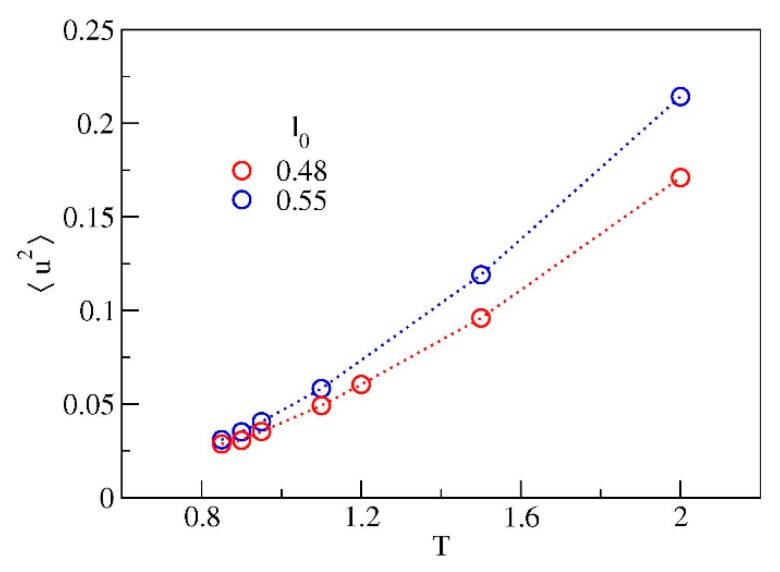
Temperature dependence of the Debye-Waller factor $\langle u^{2}\rangle$. Color code as in Figure 1.

The position of $\langle u^{2}\rangle$ is marked in Figure 6 with black circles. We remind that the DW factor provides a measure of the rattling motion of the monomer in the cage of the first neighbours. The temperature dependence of DW is reported in Figure 7. The dependence is *not* linear evidencing that, even at very short time scales, that is, $t^{*}\approx 1$ (corresponding to a few picoseconds [11], see also Reference [37]), the rattling of the monomer in the cage is *not* harmonic. Notably, the two systems exhibit different curves for $\langle u^{2}\rangle$. In particular, at fixed temperature the system with chains having longer bond length exhibits larger DW factor.

We are now in a position to test the correlation between the vibrational dynamics and the relaxation. Recently, this correlation has been examined in great detail as far as the *primary* relaxation is concerned [11]. It was found that numerical models *not providing appreciable secondary relaxations* exhibit striking agreement with experimental data of polymers where the presence of JG relaxation is known, like 1,4 Polybutadiene, Polyvinylchloride and Polymethylmethacrylate [4,6,7,38]. This finding needs clarification since the secondary relaxation acts at an intermediate time scale between the fast vibrational dynamics and the primary relaxation and it could, in principle, interfere with the correlation.

**Figure 8:**
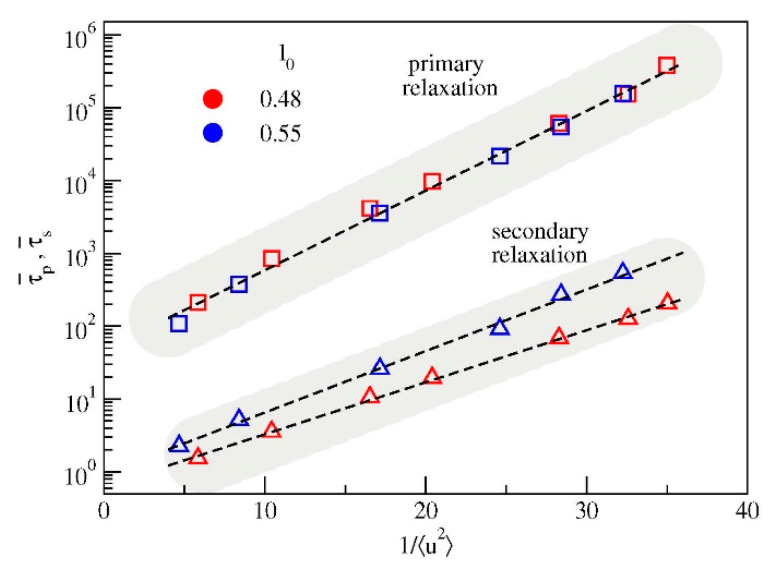
Average relaxation times ${\bar{\tau }}_{p}$ and ${\bar{\tau }}_{s}$ versus the inverse Debye-Waller factor. The correlation between the vibrational dynamics and the primary relaxation time ${\bar{\tau }}_{p}$ is unaffected by the changes of both the strength and the relaxation time of the secondary relaxation, see Figure 4. Color code as in Figure 1.

To address this aspect, Figure 8 plots the correlation between the average relaxation times and the DW factor. As major result of the present paper, it is clearly seen that the correlation between the fast vibrational dynamics and the primary relaxation is *not* affected by the presence of the secondary relaxation, in the sense that the data concerning the systems exhibiting either strong (*l*_0_ = 0.48) or weak (*l*_0_ = 0.55) secondary relaxation exhibit the same master correlation curve ${\bar{\tau }}_{p}\;\mathrm{vs.}\;\langle u^{2}\rangle$. Notably, this coincidence takes place even if the correlation curves ${\bar{\tau }}_{s}\;\mathrm{vs.}\;\langle u^{2}\rangle$ do depend on the bond length.

It seems proper to comment on a feature of Figure 8. The correlation between the primary relaxation and the DW factor of *distinct* systems is usually seen in terms of the reduced DW factor $\langle u^{2}\rangle /\langle u_{g}^{2}\rangle$ to get rid of specific system-dependent aspects, $\langle u_{g}^{2}\rangle$ being the DW factor at the glass transition temperature [11]. Figure 8 shows that the correlation of the two systems under study is rather high by considering the unscaled DW factor. This suggests that, even if the glass transition of the two systems is *different* due to the different temperature dependence of the primary relaxation times, see Figure 5 (left), the DW factor at the glass transition of the two systems is *equal* within the errors.

### 3.4. Alternative Probe Functions of Secondary Relaxation

The present study reveals the JG relaxation via the bond correlation function *C*(*t*), Equation (4). We pose the question of the JG sensitivity of alternative relaxation functions. We focus here on the familiar intermediate scattering function (ISF) [11,39] and the torsional autocorrelation function (TACF) [14]. ISF is defined as [11,39]:

(11) $$F_{s}\left(q,t\right)=\frac{1}{N}\langle \overset{N}{\underset{i=1}{\sum }}e^{iq\cdot \left(x_{i}\left(t\right)-x_{i}\left(0\right)\right)}\rangle$$

In an isotropic liquid, ISF depends only on the modulus of the wavevector $q=\left|\left|q\right|\right|$ and features the rearrangements of the spatial structure of the fluid over the length scale ∼2π/*q*. As alternative relaxation function, the segmental relaxation has been characterized by TACF [14]:

(12) $$\mathrm{TACF}\left(t\right)=\frac{1}{N_{c}}\frac{1}{M-3}\overset{N_{c}}{\underset{n=1}{\sum }}\overset{M-3}{\underset{m=1}{\sum }}\frac{\langle |\theta_{m,n}\left(t\right)||\theta_{m,n}\left(0\right)|\rangle -{\langle |\theta_{m,n}\left(0\right)|\rangle }^{2}}{{\langle |\theta_{m,n}\left(0\right)|}^{2}\rangle -{\langle |\theta_{m,n}\left(0\right)|\rangle }^{2}}$$

where $\left|\theta_{m,n}\left(t\right)\right|$ is the modulus of the *m*-th dihedral angle of the *n*-th chain at a given time *t* [40].

**Figure 9:**
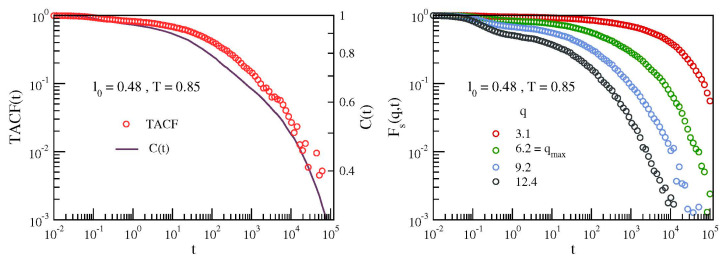
Left: comparison between the torsional autocorrelation function (TACF) and the bond-orientation correlation function, C(*t*) for the system with stronger secondary relaxation (*l*_0_ = 0.48) at the lowest investigated temperature (*T* = 0.85). Notice the step observed in both functions at $t\sim 4\cdot 10^{3}$, signalling the secondary relaxation, see Figure 2, left. The step is much more apparent in C(t), occurring when the latter is dropped of ∼40% only. The same feature is observed in TACF when more than 90% of the decay has been completed. Right: corresponding intermediate scattering function (ISF) for different wavevectors in a range including *q* = *q*_max_, corresponding to the maximum of the static structure factor. No apparent two-step decay is seen.

Figure 9 compares the bond correlation function *C*(*t*), Equation (4), with ISF and TACF at the lowest investigated temperature (*T* = 0.85, where the primary and the JG relaxation are expected to have the largest separation). A distinctive feature of *C*(*t*) at that temperature is the presence of the step at $t\sim 4\cdot 10^{3}$, signalling the secondary relaxation, see Figure 2, left. The step is clearly visible when *C*(*t*) drops ∼40% only. Figure 9, left, shows that the same step is also observed in TACF but when more than ∼90% of the decay has been completed. Namely, it is harder to be observed. Figure 9, right, plots ISF for different wavevectors in a range including *q* = *q*_max_ ∼ 2*π*/*σ*, corresponding to the maximum of the static structure factor. It is seen that there is no evidence of the two-step decay observed in the bond correlation function *C*(*t*) at the same temperature, see Figure 2, left. The results presented in Figure 9 are not unexpected. Previous MD studies [13] performed by using the same model of the present work (dubbed FRC model in Reference [13]) reported that ISF needs lower temperatures to reveal a two-step process in the relaxation. This suggests that ISF has lower JG resolution.

The ability to resolve in a limited time and temperature range both primary and secondary relaxation is related to their relative separation in time scale as well as relative strength. Concerning ISF, timescale separation is possible exploring high wavevector q, in order to be more sensitive to local and restricted dynamics, but the relative strength is also affected by this choice [41]. As recently shown [42], the microscopic density correlation function is dominated at short times by spatial fluctuations of some molecules within the cage formed by the nearest neighbours, that is, by rattling motions within the cage, while at longer times part of the relaxation strength is related to large spatial fluctuations extending at least up to the intermolecular distance, that is, outside the cage. The faster type of motion has been identified as the secondary, while the slower and more intense is the primary one. Timescale separation is usually larger for orientational dynamics, that entails for primary motions a much larger timescale than the intermediate scattering functions (as shown also in Figure 9). In contrast, for the secondary relaxation only local and fast rearrangements are probed by both observables (if the correct wavevector *q* is chosen). Again, *C*(*t*) is much more sensitive than ISF to the presence of secondary relaxation because the relative strength is dominant. Indeed, secondary processes are characterized by rare (but not negligible) and fast rearrangements, resulting in large-amplitude angle motions, which bring a considerable decorrelation of the orientation function [15]. For these reasons orientational correlation function is usually much more sensitive to detect and resolve secondary motions with respect to what happen for intermediate structure function or density autocorrelation function. This has been demonstrated by experiments and numerical simulation studies [43,44,45].

## 4. Conclusions

We have studied by MD simulations two melts of polymer chains with different bond length, resulting in rather different strength of the secondary JG relaxation. Our interest is the correlation between the fast vibrational dynamics, as sensed by the Debye-Waller factor, and the slow primary relaxation in the presence of JG relaxation. Multiple relaxation processes were searched by using the bond-orientation correlation function, *C*(*t*) which proved higher JG sensitivity with respect to alternatives provided by TACF and ISF functions. We find that changing the bond length alters both the strength and the relaxation time of the secondary relaxation, as well as the correlation with the DW factor. On the other hand, it leaves unaffected the correlation between the vibrational dynamics and the primary relaxation. This finding is in harmony with previous studies reporting that numerical models *not showing secondary relaxations* exhibit striking agreement with experimental data of polymers where the presence of JG relaxation is known [11].

The present result fits into the context of the recent debate about the universality of the correlation between fast and slow degrees of freedom. Actually, a relation between viscous flow and vibrational properties in glass-forming materials has been found in recent studies examining the fragility and the nonergodicity factor, as obtained from scattering techniques in the glassy state [46] and extrapolated to the glass transition region [47]. These two quantities were found strongly correlated only once the effect of secondary relaxation processes, if present, was correctly accounted for. With regards to this issue, it is noteworthy to mention that DW factor as defined in Equation (10) is the best definition of cage vibration amplitude, devoid of any further decorrelation due to JG relaxation.

## Abbreviations

ISF	Intermediate scattering function
JG	Johari-Goldstein
MD	molecular-dynamics
MSD	Mean square displacement
DW	Debye-Waller
